# Arctic sea ice–air interactions weaken El Niño–Southern Oscillation

**DOI:** 10.1126/sciadv.adk3990

**Published:** 2024-03-29

**Authors:** Jiechun Deng, Aiguo Dai

**Affiliations:** ^1^ Key Laboratory of Meteorological Disaster, Ministry of Education (KLME)/Joint International Research Laboratory of Climate and Environmental Change (ILCEC)/Collaborative Innovation Center on Forecast and Evaluation of Meteorological Disasters (CIC-FEMD), Nanjing University of Information Science and Technology, Nanjing 210044, China.; ^2^ Department of Atmospheric and Environmental Sciences, University at Albany, State University of New York, Albany, NY 12222, USA.

## Abstract

El Niño–Southern Oscillation (ENSO) over the tropical Pacific can affect Arctic climate, but whether it can be influenced by the Arctic is unclear. Using model simulations, we show that Arctic sea ice–air interactions weaken ENSO by about 12 to 17%. The northern North Pacific Ocean warms due to increased absorption of solar radiation under such interactions. The warming excites an anomalous tropospheric Rossby wave propagating equatorward into the tropical Pacific to strengthen cross-equator winds and deepen the thermocline. These mean changes dampen ENSO amplitude via weakened thermocline and zonal advective feedbacks. Observed historical changes from 1921–1960 (with strong sea ice–air interactions) to 1971–2000 (with weak interactions) are qualitatively consistent with the model results. Our findings suggest that Arctic sea ice–air interactions affect both the mean state and variability in the tropical Pacific, and imply increased ENSO amplitude as Arctic sea ice and its interactions with the atmosphere diminish under anthropogenic warming.

## Teaser

Interactions between Arctic sea ice and the atmosphere can dampen ENSO amplitude in the tropics.

## INTRODUCTION

El Niño–Southern Oscillation (ENSO) is the dominant mode of interannual variability over the tropical Pacific (*1*), which has far-reaching impact on both tropical and extratropical weather and climate (*2*, *3*), including the polar regions (*4*–*6*). In particular, ENSO has been identified as a main source of the interannual variability of Arctic sea ice cover (SIC) and surface air temperature (Tas) (*7*–*9*). It is suggested that ENSO-related tropical signals can propagate poleward into the Arctic by inducing anomalous atmospheric circulation (*9*, *10*) and thus modulating the Arctic Oscillation (*6*, *7*), Arctic polar vortex (*11*), and other high-latitude circulation fields (*9*, *12*). These ENSO-driven atmospheric processes can lead to changes over the Arctic (*6*, *13*), thus forming the tropical Pacific–to–Arctic teleconnection (*5*). On the other hand, observations and model simulations show that the variability and amplitude of ENSO-related sea surface temperature (SST) anomalies can be influenced by long-term transient and equilibrium warming externally induced by greenhouse gases (*14*, *15*) and modulated by extratropical internal modes such as the North Pacific Meridional Mode (*16*), Atlantic Multidecadal Oscillation (*17*), and Atlantic Meridional Overturning Circulation (*18*). Recently, the extratropical atmospheric variability (e.g., the North Pacific Oscillation) is found to influence the development of ENSO events through generating anomalous Rossby waves that propagate equatorward (*19*), implying an extratropical-to-tropical Pacific teleconnection.

Arctic sea ice insulates the underlying ocean from the atmosphere, suppressing air-sea exchange fluxes. Thus, variations and changes in SIC can lead to large changes in surface energy and water fluxes, such as increased absorption of solar radiation in summer and increased oceanic heat release in winter under low SIC conditions (*20*). Changes in upward fluxes can affect atmospheric conditions, which, in turn, alter downward radiation and surface turbulent fluxes, thereby inserting a feedback effect on sea ice. We call these processes as sea ice–air two-way interactions, which can amplify multidecadal variations in the Arctic and North Atlantic (*21*). Declining Arctic sea ice also leads to increased oceanic heating of the atmosphere during winter, amplifying Arctic warming (*20*, *22*). However, the climate response to Arctic sea ice loss is not confined to the northern high latitudes, and it can reach the tropical Pacific (*23*–*25*). Arctic sea ice loss can induce atmospheric circulation changes over the extratropical North Pacific to warm the central tropical Pacific, resulting in more frequent strong El Niño and/or central Pacific (CP) El Niño events (*24*, *25*), indicating an Arctic–to–tropical Pacific teleconnection. This raises important questions: Could the sea ice–air interactions over the Arctic have a notable impact on ENSO? And, if so, how do the sea ice–induced surface flux changes cause such an Arctic–to–tropical Pacific teleconnection? Answers to these questions have major implications for the formation mechanisms of ENSO and its future projection, as the Arctic is projected to be ice-free during the sea ice melting season in upcoming decades (*26*), which would diminish the sea ice–air interactions.

Here, we used fixed SIC in calculating surface fluxes in a fixed-ice simulation (referred to as FI), which effectively cuts off the sea ice–air two-way interactions compared with a fully coupled preindustrial control simulation (referred to as FC) using the Community Earth System Model version 1.2.1 (CESM1). Thus, the FC minus FI difference (including the SIC difference) represents the impact of sea ice–air interactions (see Materials and Methods). Using these long model simulations and historical data, we show that ENSO’s amplitude is weakened substantially when Arctic sea ice is fully coupled with the atmosphere compared with the case without the coupling, implying a substantial damping effect on ENSO by Arctic sea ice–air interactions. The weakening of ENSO is mainly driven by tropical Pacific mean-state changes that are induced remotely by an anomalous Rossby wave excited by atmospheric heating over the northern North Pacific due to these interactions. Therefore, our findings suggest that Arctic sea ice–air interactions play a notable role in modulating the tropical Pacific mean state and variability. This has major implications for future ENSO activities as Arctic sea ice and the associated sea ice–air interactions are projected to diminish under anthropogenic warming.

## RESULTS

### Weakened ENSO amplitude and underlying mechanisms

**Fig. 1.**
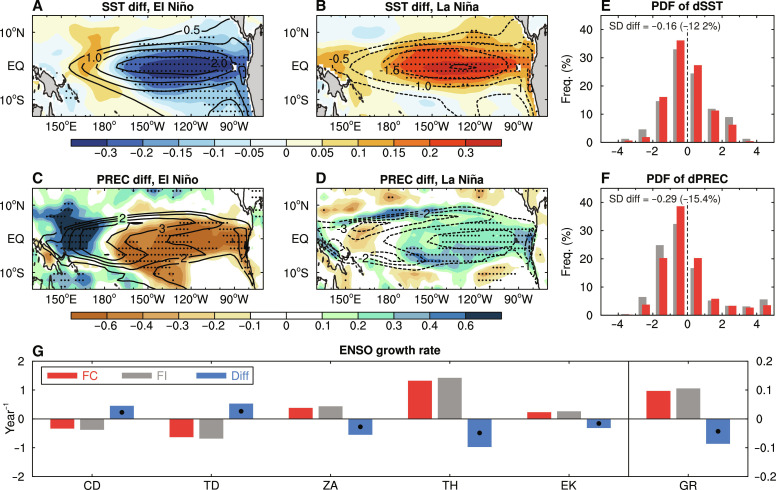
ENSO-related difference induced by Arctic sea ice–air interactions in CESM1 model. (**A** and **B**) The difference of the 7-year high pass–filtered ONDJF-mean SST anomalies (dSST; shading, in degrees Celsius) over the tropical Pacific averaged over (A) El Niño and (B) La Niña events between the FC and FI runs during years 1 to 500. (**C** and **D**) Same as (A) and (B), but for the similarly filtered ONDJF-mean precipitation (PREC) anomalies (dPREC; shading, in millimeters per day). The contours show the composite anomalies from FC. (**E** and **F**) Probability density function (PDF) of similarly filtered ONDJF-mean anomalies of (E) SST (in degrees Celsius) and (F) precipitation (in millimeters per day) averaged over the Niño3.4 region (5°S to 5°N, 170° to 120°W) in FC (red bars) and FI (gray bars) during years 1 to 500. The *x* axis is for the anomalies, and the *y* axis is for the occurrence frequency (percentage of all years). The absolute and percentage differences of the SD of anomalies in FC relative to FI are also given on the top-left corner. (**G**) The ENSO growth rate and its contributing terms (in year^−1^; see Materials and Methods) over the CEP (5°S to 5°N, 180° to 80°W) averaged from JJAS during years 1 to 500 from the FC (red bars, left *y* axis) and FI (gray bars, left *y* axis) runs and their differences (blue bars, right *y* axis; i.e., FC minus FI). The CD, TD, ZA, TH, EK, and GR represent mean current damping, thermodynamic damping, zonal advective feedback, thermocline feedback, EK feedback, and linear growth rate (the sum of all processes), respectively. EQ, equator. The dots in (A) to (D) and (G) indicate the difference is statistically significant at the 5% level based on a Student’s *t* test and a resampling technique, respectively (see Materials and Methods).

In the FC run, El Niño events have smaller anomalies of SST and precipitation in the central-to-eastern equatorial Pacific (CEP) during October to February (ONDJF) when ENSO usually matures (Fig. 1, A and C), compared to the FI run with sea ice–air interactions being cut off. Specifically, the ONDJF-mean SST and precipitation anomalies over the Niño3.4 region (5°S to 5°N and 170° to 120°W) are 0.25°C (11.7%) and 0.57 mm day^−1^ (16.6%) weaker for El Niño events in FC than in FI, respectively. The associated anomalies in zonal wind stress and subsurface ocean temperatures in the equatorial Pacific also become weaker (fig. S1, A and E) for El Niño events when sea ice–air interactions are present in the FC run. For La Niña events, these difference patterns are similar but with nearly opposite sign and slightly smaller magnitude (Fig. 1, B and D, and fig. S1, B and F), implying some asymmetric responses of ENSO-related fields (*27*).

The interannual variability as measured by SD of SSTs and precipitation over the Niño3.4 region during ONDJF is weakened by about 12 to 15% due to the sea ice–air interactions (Fig. 1, E and F). As there is little change in the ENSO period or frequency (fig. S2), this reduction in ENSO activity comes mainly from its reduced amplitude. This indicates that Arctic sea ice–air interactions can remotely dampen ENSO-associated variations over the equatorial Pacific, thus leading to fewer strong El Niño and La Niña events (with SST anomalies outside the range of ±2°C; Fig. 1E). Although there is little change in ENSO occurrence frequency as reflected by the similar number of ENSO events (80 and 79 El Niños plus 65 and 68 La Niñas in the FC and FI runs, respectively), the ratio of the number of CP El Niño events to eastern Pacific (EP) El Niño events increases by about 50% in FC relative FI (see Materials and Methods), implying that CP El Niño events would occur more frequently under strong sea ice–air interactions. Overall, these results suggest that Arctic sea ice–air interactions can weaken the amplitude of ENSO-related anomalies over the CEP substantially by 12 to 17% during ONDJF, which is unlikely to provide a noticeable feedback effect on Arctic sea ice. This dampening effect is found to be stronger during the developing and mature stages of both El Niño and La Niña events, especially from June to September (JJAS) when Arctic sea ice melts seasonally, than their other stages (fig. S3).

**Fig. 2.**
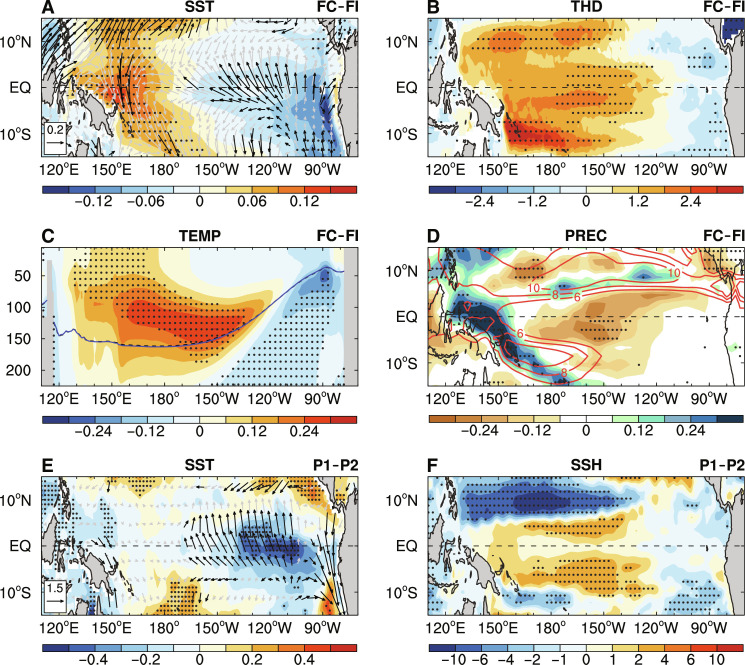
The mean-state difference over the tropical Pacific based on CESM1 and observations. The FC minus FI difference of JJAS-mean (**A**) SST (shading, in degrees Celsius) and surface winds (vectors; in meters per section), (**B**) thermocline depth (THD; measured by the depth of 20°C isotherm; shading, in meters), and (**D**) precipitation (PREC; shading, in millimeters per day) during years 1 to 500. The red contours in (D) denote climatological mean precipitation in FC. (**C**) Same as (A), but for JJAS-mean ocean temperature difference (TEMP; shading, in degrees Celsius) averaged over the equatorial Pacific (5°S to 5°N) as a function of longitude and depth. The blue solid and black dashed contours in (C) denote the 20°C isotherm in FC and FI, respectively. (**E** and **F**) The multidecadal differences of JJAS-mean anomalies (with the forced component removed; see Materials and Methods) of (E) SST (shading, in degrees Celsius) and surface wind stress (vectors, 10^−2^ N m^−2^) and (F) sea surface height (SSH; shading, in centimeters) between 1921–1960 (P1, with strong interactions and weak ENSO activity) and 1971–2000 (P2, with weak interactions and strong ENSO activity) (i.e., P1 minus P2). The black vectors in (A) and (E) and the stippling in all panels indicate that the difference is statistically significant at the 5% level based on a Student’s *t* test.

To reveal the mechanisms underlying the weakening of ENSO amplitude, we further analyze the ENSO growth rate based on the Bjerknes stability index (BJ index), a widely used measure of ENSO’s growth rate (see Materials and Methods) (*28*), over the eastern equatorial Pacific region during JJAS before ENSO peaks. We found that the ENSO growth rate is reduced by 0.09 year^−1^ (~8.5%) in FC relative to FI (Fig. 1G), indicating that sea ice–air interactions decrease the ENSO growth rate during JJAS and thus result in a weak ENSO event, consistent with the reduced ENSO amplitude. Among all the contributing factors (Fig. 1G), a substantial decrease (−0.1 year^−1^) in the thermocline (TH) feedback plays a dominant role in weakening ENSO, followed by the decreased zonal advective (ZA) feedback (−0.06 year^−1^) and Ekman (EK) feedback (−0.03 year^−1^), and they are partly offset by the reduced (i.e., weaker) damping effect from mean currents and thermodynamic processes. This means that Arctic sea ice–air interactions weaken ENSO amplitude primarily through two previously known processes that can affect ENSO variability (*17*, *29*, *30*): deepening the mean thermocline over the central-to-western equatorial Pacific and increasing the zonal gradient of background SSTs in the equatorial Pacific (Fig. 2, A and B), which may be associated with mean trade wind changes (*28*–*31*).

To understand these ENSO growth rate changes, we examine the tropical Pacific mean-state differences caused by the sea ice–air coupling, focusing on JJAS. The climatological trade winds, which feature strong cross-equator southeasterly winds in the central-eastern tropical Pacific (fig. S4A), are markedly intensified by sea ice–air interactions, with surface southeasterly wind anomalies extending from the southeastern to northeastern tropical Pacific (Fig. 2A). Accordingly, more warm surface waters would pile up over the central-western Pacific (*32*) with strong warmer anomalies at the depth of about 100 to 200 m (Fig. 2C) due to the enhanced mean easterly wind. Thus, the mean thermocline is substantially deepened (although with small magnitude) over the central-western tropical Pacific (Fig. 2, B and C). This deepening can dampen the TH feedback as it reduces the thermocline's sensitivity to wind stress anomalies during ONDJF (*17*, *29*, *33*); that is, ENSO-related zonal wind stress anomalies (also weakened) in the west cannot produce large subsurface ocean temperature anomalies over the equatorial Pacific (fig. S1, C to F). Meanwhile, the strong subsurface ocean warming over the CP also decreases the upper-ocean stratification (fig. S4B), which can dampen the ocean response to a wind stress forcing (*15*) and, thus, the EK feedback. These suppressed positive feedbacks ultimately weaken ENSO growth and its amplitude.

On the other hand, the strengthened mean southeasterly winds in the central-eastern tropical Pacific also increase the surface evaporation and thus latent heat release from the ocean to the atmosphere (fig. S4C) to cool SSTs there (Fig. 2A), namely, through the wind-evaporation-SST (WES) feedback (*34*). These colder SSTs are found to be stronger to the south of the equator than to the north in the EP, especially near the South American coast, which is likely due to the enhanced local upwelling (fig. S4D) under stronger mean trade winds. Thus, the south-north SST gradient is enlarged in the EP, and this would further increase the mean trade winds and, thus, colder SSTs there through the WES feedback. Similarly, the mean SSTs become warmer in the western tropical Pacific due to weakened local mean winds and decreased surface upward latent heat flux (fig. S4C). Warmer mean SSTs in the western tropical Pacific and colder mean SSTs in the east increase background west-minus-east zonal SST contrast in the tropical Pacific and thus enhance the mean easterly wind. In addition, the mean precipitation is also moderately suppressed over the central equatorial Pacific (Fig. 2D) due to sea ice–air interactions. Because of these mean-state changes, ENSO-related SST anomalies cannot effectively cause large precipitation anomalies over the CEP (Fig. 1, C and D) (*33*), together with weak zonal wind stress anomalies in the west and thus weakened ZA feedback (fig. S1, A and B), leading to weak ENSO activity.

In summary, the intensified climatological trade winds over the CEP induced by sea ice–air interactions play a crucial role in weakening ENSO amplitude mainly through dampening the TH and ZA feedbacks. Our results are consistent with previous findings that ENSO would become weaker under stronger mean trade winds due to reduced TH and/or ZA feedbacks (*17*, *29*, *33*). Below, we will show that these tropical mean-state changes originate from anomalous warming over the northern North Pacific induced by sea ice–air interactions.

Using observational and reanalysis data, we also found that ENSO activity increased from 1921–1960 (period 1 or P1) to 1971–2000 (period 2 or P2). Although this increased ENSO activity can be caused by the background global warming (*15*, *35*), the correlation of ENSO activity with the externally forced global mean Tas is not overly strong (with a correlation of 0.33, *P* < 0.01; fig. S5). This implies that anthropogenic warming may only have played a partial role (accounting for about 11%) in driving these historical changes, and internally generated multidecadal changes are likely a major contributor. We found that SIC variations decreased from P1 to P2 along the marginal ice zones (MIZ) over the Pacific sector of the Arctic during JJAS, e.g., the Bering-Okhotsk Sea (BOS) region (fig. S5), with a correlation of −0.73 (*P* < 0.01) between the variability of ENSO and BOS SIC, implying a significant connection between the two. Note that the strong SIC variations during P1 may imply enhanced sea ice–air interactions, as these interactions greatly amplify the variability of SIC, surface albedo, surface heat fluxes (fig. S6, A to D), and surface temperature (*21*), while a constant SIC would represent no two-way interactions, which is the case in the FI run. Thus, the P1 minus P2 difference can be used as a measure of the impact from the stronger sea ice–air interactions in P1 relative to P2, qualitatively resembling the FC (with the sea ice–air coupling) minus FI (without the two-way coupling) difference.

The multidecadal differences between these two periods (i.e., P1 minus P2) show that the weak ENSO amplitude during P1 is accompanied by strengthened background winds with colder SSTs in the EP and thus enhanced climatological zonal SST gradients over the equatorial Pacific and a deepened thermocline in the central tropical Pacific (Fig. 2, E and F), similar to the CESM1 FC minus FI differences (Fig. 2, A and B). Although these multidecadal differences in the real world could be caused by many factors, their association with the SIC variability change suggests that these results are qualitatively consistent with our CESM1 results and implies that Arctic sea ice variability (as a measure of sea ice–air interaction strength) could modulate ENSO activity, although it is difficult to disentangle the cause-and-effect relationship in the fully coupled real world.

### Mean-state changes over the Arctic and high-latitude North Pacific

**Fig. 3.**
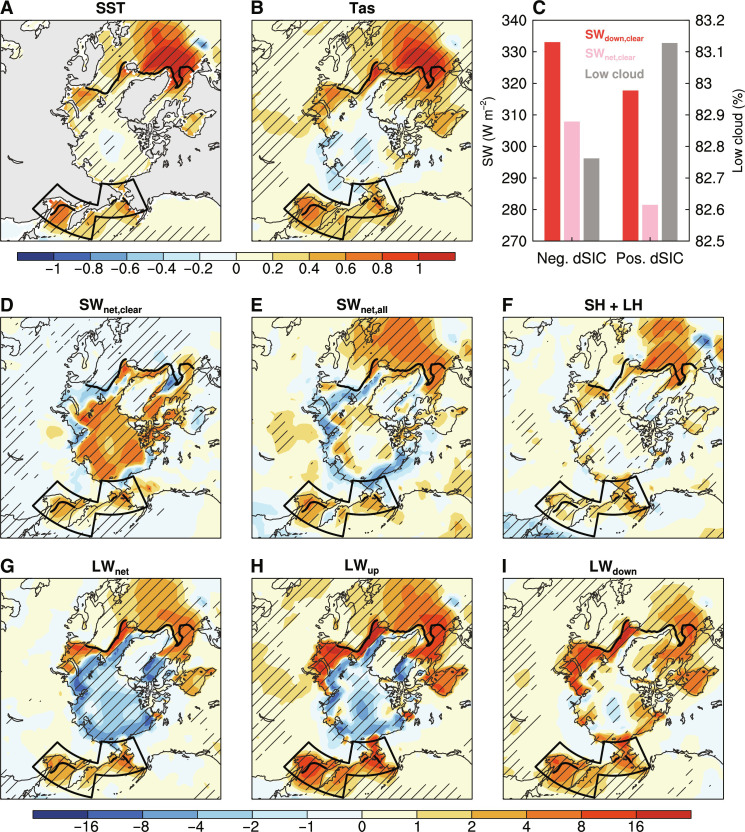
CESM1-simulated differences of temperature and surface energy flux over the Arctic. (**A** and **B**) The FC minus FI differences of JJAS-mean (A) SST (in degrees Celsius) and (B) Tas (in degrees Celsius) north of 50°N during years 1 to 500. (**C**) Composites of JJAS-mean clear-sky surface downward (SW_down_; red bars) and net (SW_net_; pink bars) SW radiation (in watts per square meter, left *y* axis) and low cloud amount (gray bars; in percentage, right *y* axis) over the grid boxes of the BOS [outlined in (A)] with JJAS-mean SIC > 0 in FC averaged over years with the negative and positive SIC anomalies (dSIC; relative to the FC mean) outside the ±1.5 SD range. (**D** to **I**) Same as (A) and (B), but for (D) clear-sky and (E) all-sky surface SW_net_, (F) turbulent (sensible plus latent) heat flux (SH + LH), and surface (G) net (LW_net_), (H) upward (LW_up_) and (I) downward (LW_down_) LW radiation. All the surface radiation and heat flux are in watts per square meter. Note that SW_down_, SW_net_, and LW_down_ are positive downward, and LW_net_, LW_up_, and SH + LH are positive upward. The outlined areas in each panel [except (C)] define the BOS region within 170°E to 155°W and 58° to 70°N for the Bering Sea and 135° to 170°E and 50° to 62°N for the Okhotsk Sea, and the black contour denotes the climatological JJAS-mean SIC edge (for SIC = 5%) from FC. The hatching indicates the difference is statistically significant at the 5% level based on a Student’s *t* test.

The above results highlight the important role of sea ice–air interactions in causing tropical mean-state changes relevant to ENSO formation. Here, we examine the mean-state changes in and around the Arctic. Sea ice–air interactions substantially increase the mean SST and Tas along the MIZ during JJAS (Fig. 3, A and B), especially over the BOS region and the subpolar North Atlantic, whereas little change is seen over the central Arctic.

Although the atmosphere “sees” similar mean SIC in the two runs by design (see Materials and Methods), the positive and negative SIC anomalies appear to have different impacts on shortwave (SW) radiation in the FC run: The negative SIC anomalies are associated with higher clear-sky surface downward (and net) SW radiation and fewer low clouds than the positive SIC anomalies (Fig. 3C). This implies that lower SIC tends to occur during relatively sunny and warm conditions (e.g., during daytime or sunny days), which allows the SIC decrease to increase surface SW absorption greatly. In contrast, higher SIC mainly occurs under cloudy and cool conditions (e.g., at night or during cloudy days) with low incoming surface SW radiation, which results in a smaller amount of reduction in surface absorbed SW radiation. As a result, the SIC variations from sea ice–air interactions in the FC run substantially increase the mean surface absorbed SW radiation averaged over both negative and positive SIC anomalies in comparison to the fixed SIC case in the FI run (Fig. 3D). Thus, the different occurrence timing of the positive and negative SIC anomalies from the SIC variations in FC can lead to a mean change in absorbed surface SW radiation relative to FI. Meanwhile, these interactions also moisten the lower troposphere and thus increase low cloud amount over the central Arctic (fig. S7, A and B), which greatly dampens the increased surface SW absorption there (Fig. 3, D and E), although both are triggered by sea ice–air interactions. Thus, the increase in surface net downward SW radiation is confined only to the MIZ, especially over the BOS region and the subpolar North Atlantic (Fig. 3E), leading to warmer mean SST there (Fig. 3A). This increased surface temperature (Fig. 3A and fig. S7C) slightly enhances turbulent heat fluxes into the air (Fig. 3F). Furthermore, the local surface-air temperature gradient is enlarged (fig. S7D), which increases (net) upward longwave (LW) radiation (Fig. 3, G and H) to cause warmer Tas over the North Pacific and North Atlantic high latitudes (Fig. 3B). The resultant warmer air would, in turn, increase downward LW radiation (Fig. 3I) to further warm the surface over the BOS region and the subpolar North Atlantic.

**Fig. 4.**
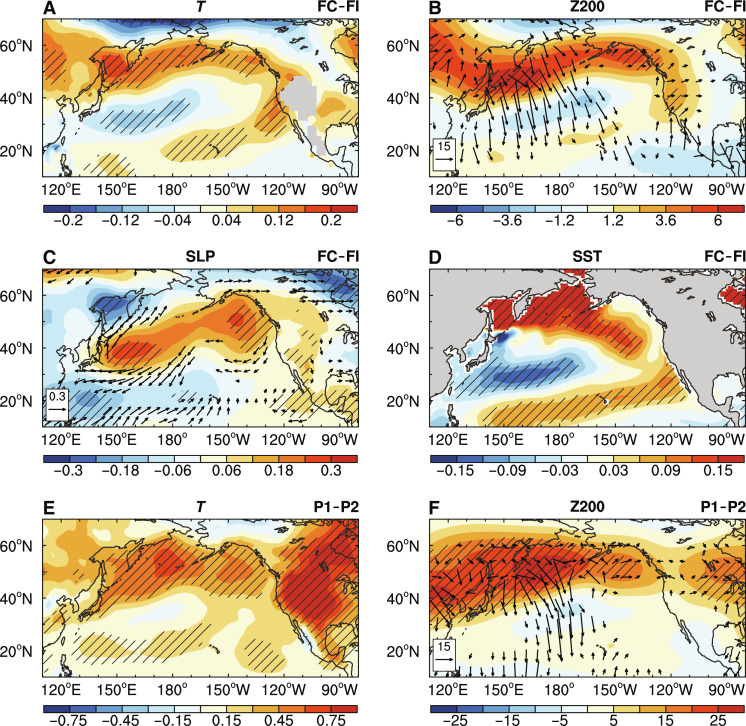
Physical processes for the Arctic–to–tropical Pacific teleconnection. (**A** to **D**) The FC minus FI differences of JJAS-mean (A) air temperature (*T*; shading, in degrees Celsius) averaged over 1000 to 850 hPa, (B) 200-hPa geopotential height (Z200; shading, geopotential meters) and the corresponding wave activity flux (vectors, 10^−3^ m^2^ s^−2^), (C) SLP (shading, in hectopascals) and surface winds (vectors, in meters per second), and (D) SST (in degrees Celsius) over the North Pacific north of 10°N during years 1 to 500. (**E** and **F**) The multidecadal differences of the JJAS-mean (E) 1000- to 850-hPa *T* (in degrees Celsius) and (F) Z200 (shading, geopotential meters) and the wave activity flux (vectors, 10^−2^ m^2^ s^−2^) between P1 and P2 (i.e., P1 minus P2) based on 20th Century Reanalysis version 3 (20CRv3). Note that only vectors with magnitude greater than 3 × 10^−3^ and 3 × 10^−2^ m^2^ s^−2^ are shown in (B) and (F), respectively. The black vectors in (C) and the hatching in all panels indicate the difference is statistically significant at the 5% level based on a Student’s *t* test.

A surface energy budget analysis (see Materials and Methods) over the BOS region further reveals that surface net upward energy is increased by 1.74 W m^−2^ during JJAS mainly due to the increase in net upward LW radiation (1.94 W m^−2^) and turbulent heat flux (1.14 W m^−2^) initiated by more SW absorption at the surface (1.34 W m^−2^) under sea ice–air interactions as discussed above. This leads to lower tropospheric warming over the BOS (Fig. 4A), which is caused by increased SST because of enhanced surface absorption of SW radiation during JJAS induced by the sea ice–air coupling in FC relative to FI.

Associated with the observed weak ENSO activity during P1 relative to P2, enhanced surface net upward fluxes of energy also appeared along the MIZ over the BOS (fig. S6E), which could be partly caused by the stronger sea ice–air interactions during P1 (fig. S6, A to D). We should note that this increased surface net upward energy comes mainly from the increased net upward SW radiation there (fig. S6, E to G), rather than the enhanced net upward LW radiation and turbulent heat fluxes as in our CESM1 simulations. As a result, there is more SW radiation absorbed by the atmosphere (fig. S6H) that would cause lower tropospheric warming over the northern North Pacific during P1 (Fig. 4E). This indicates that anomalous heating along the MIZ over the Bering Sea in P1 (relative to P2) and FC (relative to FI) may result from different surface heat flux changes induced by sea ice–air interactions, possibly due to differences in the sea ice–air coupling between the reanalysis, which was generated by using specified SST and SIC from observations, and the fully coupled CESM1 FC run, which has two-way surface interactions.

### Arctic–to–tropical Pacific teleconnection

How would the above mean-state changes over the Arctic and northern North Pacific (mainly the BOS region) cause remote mean-state changes over the tropical Pacific? During JJAS, the increased surface net upward energy over the BOS in FC due to the sea ice–air coupling causes tropospheric warming that is largest near the surface and extends to ~200 hPa (Fig. 4A and fig. S8A), which leads to a positive 200-hPa geopotential height (Z200) anomaly (Fig. 4B and fig. S8B) and a negative sea level pressure (SLP) anomaly over the BOS (mainly the Sea of Okhotsk) (Fig. 4C) and thus ascending anomalies there (fig. S8C). The concurring anomalous divergence in the upper troposphere over most of the BOS region can locally generate a negative Rossby wave source (RWS) through the vortex squeezing (see Materials and Methods and fig. S9) and, thus, an anomalous Rossby wave propagating equatorward into the tropical Pacific. Concurrently, decreased air temperature (*T*) and Z200 with increased SLP are seen over a southwest-northeast zone just south of the BOS, while a negative SLP anomaly is located along ~20°N with weak positive Z200 anomaly (Fig. 4, A to C), featuring a northward tilted structure of such an anomalous Rossby wave (fig. S8B). Note that the increased SLP over the midlatitude North Pacific due to sea ice–air interactions is nearly opposite to that induced by Arctic sea ice loss (i.e., a negative SLP anomaly) noticed previously (*24*, *25*), implying their competing effects on the tropical Pacific mean state and, thus, ENSO variability. The above *T* changes over the North Pacific north of ~20°N reduce the south-north *T* gradient and thus upper-level zonal wind along 40°N (fig. S8D), which decelerates the southern part of the mean westerly jet to facilitate the southeastward propagation of the anomalous Rossby wave.

Along with increased SLP over the midlatitude North Pacific, the anticyclonic surface circulation anomaly extends from the east of Japan to the west of North America (Fig. 4C). On one hand, the southwesterly wind anomaly on its northwestern side can transport more warm and moist airmass poleward (fig. S7E) to further warm the SSTs in the BOS region through increased downward LW radiation (Fig. 3I); on the other hand, the northeasterly wind anomaly in the south would strengthen the prevailing trade wind along ~30°N over the North Pacific (Fig. 4C), thus causing cooler SSTs there (Fig. 4D) via the WES feedback (*34*). Meanwhile, anomalous cyclonic circulation is found in the western tropical Pacific along ~20°N together with a negative SLP anomaly there (Fig. 4C) and anomalous subsidence to its south near ~10°N (fig. S8C). Thus, the anomalous southwesterly wind along its southern flank (north of 10°N) could weaken the prevailing northeasterlies (Fig. 4C), while the anomalous northerly wind to its south (south of 10°N) decreases the mean cross-equator southerlies in the western equatorial Pacific (Fig. 2A). These mean surface wind changes ultimately lead to warmer mean SSTs near the warm pool (Fig. 4D) via the WES feedback. This increases zonal SST gradients that strengthen mean trade winds over the central-eastern tropical Pacific to induce colder SSTs in the east (Fig. 2A), which would, in turn, further intensify surface winds and thus form a positive feedback loop (i.e., the Bjerknes feedback). Such an SST response pattern over the tropical and North Pacific resembles that induced by winter SIC anomalies over the Sea of Okhotsk through increased surface heat fluxes (*36*), although they appear in different seasons. In addition, the concurring negative anomalies of wind stress curl (fig. S4E) also deepen the thermocline over the central tropical Pacific via Ekman pumping. These mean changes in equatorial trade winds and thermocline depth can substantially weaken ENSO amplitude through dampening the TH and ZA feedbacks as discussed above. The southeastward propagating anomalous Rossby wave (Fig. 4B), excited by anomalous heating over the BOS and northern North Pacific due to sea ice–air interactions, serves as a key bridge that connects the Arctic and tropical Pacific mean-state changes.

**Fig. 5.**
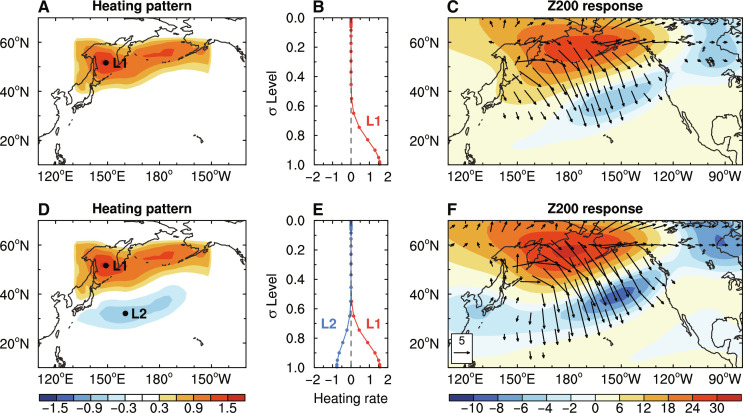
Atmospheric responses to lower-tropospheric heating simulated by LBM. (**A**) Spatial distribution of atmospheric heat forcing (in kelvin per day) used in LBM to represent the lower tropospheric warming over the North Pacific high-latitudes induced by sea ice–air interactions (Fig. 4A) and (**B**) the vertical profile of the heating rate (in kelvin per day) in sigma (σ) levels at location 1 (L1; near 52°N and 149°E) with the maximum warming. (**C**) The response of Z200 (shading, geopotential meters) to the heat forcing in (A) and its associated wave activity flux (vectors, 10^−2^ m^2^ s^−2^; only shown with magnitude greater than 1 × 10^−2^ m^2^ s^−2^). (**D** and **E**) Same as (A) and (B), respectively, but with additional tropospheric cooling over the subtropical North Pacific to further present air-sea interaction feedback in the region (Fig. 4A), with the maximum cooling at location 2 (L2; near 32°N and 160°E). (**F**) Same as (C), but for the Z200 response to the heat forcing in (D) and the related wave activity flux. Note the smaller scale for negative values in (C) and (F) for clarity.

To confirm the formation of such a teleconnection anomaly pattern, we also use a linear baroclinic model (LBM) to simulate mean-state responses to anomalous heating resembling that induced by sea ice–air interactions over the North Pacific (see Materials and Methods). With the imposed tropospheric warming (mainly in the low level) over the northern North Pacific resembling that in FC induced by the sea ice–air coupling (Fig. 5, A and B), Z200 shows strong anticyclonic circulation response over the northern North Pacific that propagates equatorward but with weak cyclonic response to its south (Fig. 5C). When the tropospheric cooling over the subtropical North Pacific (due to local air-sea interactions) is also included (Fig. 5, D and E), the cyclonic Z200 response becomes stronger in the subtropical North Pacific (Fig. 5F). These responses are similar to the Z200 difference between the FC and FI runs (Fig. 4B). Thus, the LBM modeling confirms that the anomalous Rossby wave, which connects the mean-state changes between the high-latitude North Pacific and tropical Pacific, originates from the tropospheric heating over the northern North Pacific induced by sea ice–air interactions and is enhanced by the subtropical cooling over the North Pacific.

Consistent with the CESM1 results, historical data show that the increased surface upward energy over the BOS during P1 also leads to strong lower tropospheric warming over the northern North Pacific relative to P2 (Fig. 4E). This warming can increase Z200 and generate an anomalous upper-tropospheric Rossby wave that propagates into the tropical Pacific (Fig. 4F), forming an Arctic–to–tropical Pacific teleconnection similar to that caused by sea ice–air interactions in our CESM1 simulations (Fig. 4B). These Arctic–to–tropical Pacific changes ultimately weaken ENSO amplitude during P1 through the mechanisms analyzed above for the model simulations (Fig. 2, E and F). Again, we should note that it is difficult to clearly isolate the impact of sea ice–air interactions in the real world, and these historical changes can be caused by many other factors, but these results are at least qualitatively consistent with those seen in our FC and FI runs. Thus, the relatively weak ENSO amplitude during P1 might be partly caused by the anomalous tropospheric warming over the northern North Pacific tied to the increased surface net upward energy flux over the BOS (likely due to stronger sea ice–air interactions) through the Arctic–to–tropical Pacific teleconnection described above.

## DISCUSSION

**Fig. 6.**
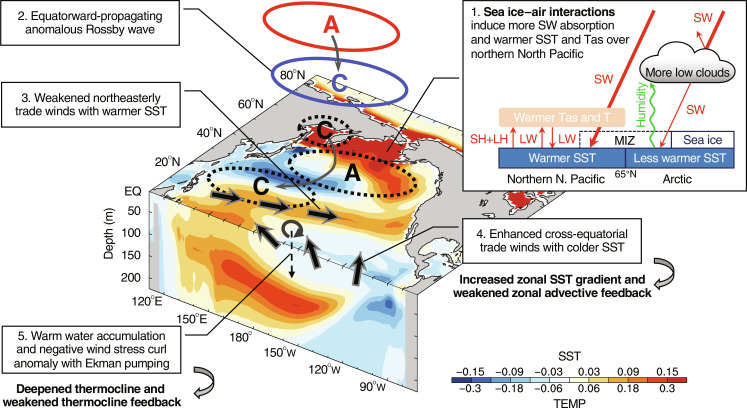
Schematic diagram for the Arctic–to–tropical Pacific teleconnection during JJAS. The longitude-latitude and longitude-depth panels are the same as Figs. 4D and 2C, respectively. The key processes shown here include the following: (1) Sea ice–air interactions increase surface SW absorption over the northern North Pacific near the MIZ (indicated by dashed lines), but with little SW changes over the central Arctic due to the damping effect of increased low clouds there, leading to strong warming confined to the northern North Pacific (mainly the BOS region); (2) the resultant anomalous atmospheric heating over the BOS excites an anomalous Rossby wave propagating equatorward (gray curve arrows) into the tropical Pacific, with an anticyclone response (labeled as A) over the northern North Pacific and a cyclonic response (labeled as C) over the subtropical Pacific in the upper troposphere (colored solid circles), and the corresponding circulation response at the surface (black dashed circles) is nearly the opposite to that at 200 hPa; (3) the associated low-level southwesterly wind anomaly (solid black arrows between 10° and 20°N) weakens the subtropical mean northeasterly trades and leads to warmer SSTs over the western tropical Pacific and thus increased zonal SST gradients in the equatorial Pacific; (4) the resultant stronger mean trade winds (solid black arrows along the equator) cause colder SSTs in the eastern tropical Pacific that further enlarge zonal SST gradients and thus weaken the ZA feedback; (5) the thermocline over the central-western tropical Pacific is deepened because of the accumulation of warm waters under stronger mean trade winds and the negative anomalies of surface wind stress curl (black circular) through Ekman downwelling (dashed black downward arrows) and thus weakened TH feedback. These processes ultimately weaken ENSO activity.

In summary, our model experiments reveal that Arctic sea ice–air interactions can remotely weaken ENSO’s amplitudes and associated variability in other fields in the tropical Pacific by 12 to 17% by modulating the tropical Pacific mean state. The main processes, summarized in Fig. 6, are as follows. During JJAS, sea ice–air interactions would lead to enhanced surface SW absorption, warmer SST, and increased upward energy fluxes over the northern North Pacific (mainly the BOS region) near the MIZ, causing anomalous warming from the surface to around 200 hPa there. Such a heating anomaly forces an anomalous cyclone at the surface over the BOS and an anticyclonic anomaly in the upper troposphere locally; it also excites an anomalous atmospheric Rossby wave propagating equatorward into the tropical Pacific. The Rossby wave creates alternating SLP patterns toward the tropical Pacific, with an anticyclonic response in the midlatitude North Pacific and a cyclonic anomaly around 20°N in the western-central Pacific. The anomalous southwesterly winds over the western-central tropical Pacific act to weaken the mean northeasterly trades, leading to warmer SSTs in the western-central tropical Pacific via the WES feedback and, thus, the enlarged zonal SST gradients over the tropical Pacific. This causes stronger mean trade winds and colder SSTs in the CEP through both Bjerknes and WES feedbacks, together with a slightly deeper mean thermocline in the western-central tropical Pacific by an accumulation of warm waters and Ekman pumping there. As a result, the ENSO amplitude is dampened because of the weakened TH feedback by the deeper mean thermocline and the weakened ZA feedback by the enlarged zonal SST gradient under stronger mean trade winds.

Historical changes from P1 (with weak ENSO, strong SIC variations, and likely strong sea ice–air interactions) to P2 (with strong ENSO, weak SIC variations, and likely weak sea ice–air interactions) based on observational and reanalysis data are qualitatively consistent with the model results, such as the surface heat flux–induced teleconnection between the Arctic and tropical Pacific. The historical changes between P1 and P2 appear to be linked to changes in the strength of the sea ice–air interactions over the Pacific sector of the Arctic. Compared with P1, ENSO activity has already strengthened in P2 when the SIC variations become much weaker during JJAS (implying a weakening of the sea ice–air interactions) (fig. S5), although many other factors may also play a role in the real world. Note that the SIC variations have also become relatively stronger after the 1990s (but much weaker than P1 due to large sea ice loss). This implies that the associated stronger sea ice–air interactions might lead to weakened ENSO amplitude and increased CP El Niño occurrence in recent decades (*24*, *31*), which is qualitatively consistent with our model results. However, this observed multidecadal relationship of the SIC variations with ENSO activity and diversity needs further investigation in multiple coupled climate models.

Our results suggest asymmetric impacts of the SIC anomalies on surface SW radiation under the sea ice–air interactions, which plays a crucial role in causing the mean-state changes over the BOS and other Arctic regions. However, the monthly model data available to this study limit our ability to further disentangle such an asymmetry in the impact of SIC on surface SW radiation between daytime and night or between sunny and cloudy days. We plan to further investigate this potentially important phenomenon related to sea ice’s impacts on our climate using high-frequency (e.g., hourly or daily) reanalysis and model data over the BOS and other MIZ.

Last, ENSO is projected to strengthen in future warmer climates by many coupled climate models (*15*, *37*), with an intensification of the ENSO-related teleconnections such as the Pacific–North American pattern (*38*, *39*). In particular, strong El Niño events would occur more frequently because of large Arctic sea ice loss (*25*), which represents a lack of or weakening in sea ice–air interactions. Our results imply that a substantial portion of the strengthened ENSO activity in future warmer climates may result from the weakened sea ice–air interactions as Arctic sea ice disappears during the warm season. That is, if the Arctic becomes seasonally ice-free in the upcoming decades (*26*), then ENSO activity will likely be amplified as the sea ice–air interactions and the associated damping effect reported above diminish, which may have implications for the strengthened Pacific–North American pattern in the future. This is in addition to the impacts of Arctic sea ice loss and the resultant warming (*24*, *25*); that is, as the ice-air interactions weaken with diminishing sea ice, the two effects (from the mean sea ice loss and ice-air interactions) should both lead to stronger ENSO. The ENSO’s close link to Arctic sea ice–air interactions highlights the need to examine more closely the impact of the diminishing sea ice on ENSO under anthropogenic warming. This requires coupled climate models to realistically simulate the internal interactions among sea ice, ocean, and the atmosphere, which may help reduce the intermodel spread in future ENSO projections (*37*, *40*).

## MATERIALS AND METHODS

### Observational and reanalysis data

To check whether any of our model results are qualitatively consistent with the real world, we analyzed historical changes between 1921–1960 (P1; with weak ENSO activity) and 1971–2000 (P2; with strong ENSO activity) using observational and reanalysis data. Monthly mean sea surface height (SSH) and surface wind stress from 1900 to 2008 were derived from the Simple Ocean Data Assimilation (SODA) on 0.5° grids (*41*). Monthly mean atmospheric fields were obtained from the 20th Century Reanalysis version 3 (20CRv3) from 1900 to 2014 on 1° grids (*42*). The 20CRv3 is the latest long-term reanalysis product generated by an atmospheric general circulation model (AGCM) forced by observed SST, SIC, and surface pressure data. For data consistency, we also used monthly mean SST and SIC data during 1900–2014 within the 20CRv3 reanalysis, which were derived from observations (namely, SODAsi.3 and HadISST2 datasets) (*42*) and used as ocean boundaries of the AGCM to produce atmospheric fields in 20CRv3.

As the externally forced global warming only accounts for about 11% of the historical change of ENSO activity during 1910–2003 (fig. S5), here, we focus on the multidecadal mean-state differences (after removing the forced changes) between P1 and P2, which should be generated by internal processes such as sea ice–air interactions discussed in this study. The externally forced component in the analyzed fields was removed from observations and reanalysis before further analyses using a linear regression method used previously (*21*, *43*). We used the anomaly time series of the global mean Tas averaged over 25 all-forcing historical runs from 25 Coupled Model Intercomparison Project phase 6 (CMIP6) (*44*) models during 1900–2014 as the forced signal (*x*), used linear regression between *x* and the anomaly time series of a given variable (such as SST or SSH as variable *y*) at each grid box to obtain the externally forced component in variable *y*, and then subtracted it from the original *y* time series before further analyses.

### CESM1 simulations

We used a fully coupled model, namely, the CESM1 (*45*) from the (NCAR) National Center for Atmospheric Research, with the Community Atmosphere Model version 4 (CAM4) as its atmospheric component. The CESM1 has been shown to reasonably simulate the mean climate and variability over both the Arctic and tropics, and it has been widely used to investigate Arctic (*21*, *22*, *46*, *47*) and tropical climate (*31*, *40*) and their teleconnection (*25*). The CESM1 used in this study has a grid increment of 2.5° × ~2.0° (longitude × latitude) for CAM4 and ~1.0° × ~0.5° (longitude × latitude) for the sea ice and ocean models.

We conducted two 500-year CESM1 preindustrial control simulations with CO_2_ fixed at 284.7 parts per million by volume (ppmv), including a fully coupled preindustrial control run (referred to as FC) with dynamic sea ice and a sensitivity fixed-ice run (referred to as FI), which is same as the FC run except that fixed SIC is used only in the coupler of the model for determining the area weights of ice and water surface types in calculating the mean fluxes for grid boxes north of 30°N. This fixed-ice setup used here has already been described in detail and used in our previous studies (*21*, *22*, *47*, *48*).

We emphasize that SIC or mass inside the model was not fixed or altered by us, only the ice fractional weighting in the coupler was fixed, which only affects the box mean values of the exchange fluxes (grid box mean flux = ice fraction × flux over ice + water fraction × flux over water) and has no effects on the ice mass or energy conservation. In addition, the calculation of the fluxes over ice or water surfaces was not altered by us. The fixed SIC was derived from the FC monthly climatology. Thus, the atmosphere should see similar mean SIC (and thus albedo) in the two runs, but the atmosphere sees the same mean SIC all the time (except for the mean seasonal cycle) through the coupler in the FI run instead of the time-varying SIC in the FC run. Our tests showed that linearly interpolating the monthly SIC climatology to daily SIC resulted in a small (0.0 to −0.4%) negative bias in the resultant monthly SIC climatology, which is an order of magnitude smaller than the SIC difference shown in fig. S7F for most of the Arctic regions. Furthermore, the SIC interpolation bias is negative, while the SIC difference over the BOS region is positive. Thus, the interpolation error is not a major source of the SIC difference (fig. S7F). In the FI run, our intervention was only applied to the coupler of the model, and we did not attempt to override or alter SIC or any other fields inside the sea ice model. That is, any sea ice–related processes (including sea ice itself and fluxes over sea ice) inside the sea ice model are still allowed to evolve dynamically and can also respond freely to any changes from the atmosphere or ocean induced by this intervention, in contrast to previous studies (*49*, *50*).

While the use of fixed SIC may alter the surface exchange fluxes between the atmosphere, ocean, and sea ice reservoirs, it does not lead to energy loss or creation as any reduced (increased) flux will allow more (less) energy to stay in the original reservoir. The same applies to water exchange fluxes. For example, if the upward energy flux is reduced by 5 W m^−2^ because of the use of fixed SIC compared with using internal SIC, then that 5 W m^−2^ of energy will remain in the surface reservoir (i.e., fractional ocean or sea ice surface depending on the SIC used in the coupler), and the atmosphere will receive 5 W m^−2^ less energy compared with the case using internal SIC. Thus, our fixed-ice setup does not alter any physical laws such as the conservation of mass, momentum and energy between the atmosphere and underlying ice and ocean surfaces. To some degree, it is similar to changing a land surface type (e.g., from natural forest to cropland), using a fixed land cover type (instead of a time-varying vegetation cover as in the real world) over a region, or using climatological SST (instead of time-varying SST) in an AGCM simulation. Although the use of this land-surface type or SST may alter the exchange fluxes and thus affect the climate, it does not violate the energy and mass conservation laws as the altered exchange fluxes do not lead to loss or creation of energy or mass among the reservoirs. The same occurs in our FI run: The use of fixed SIC alters the exchange fluxes, but it does not lead to loss or creation of energy of mass.

By using a fixed SIC in the coupler, instead of using its internal values as in the FC run, we were able to effectively cut off the two-way interactions between the atmosphere and sea ice caused by SIC fluctuations. Thus, any differences (including the SIC difference; fig. S7F) between the FC and FI runs (i.e., FC minus FI) are triggered by the sea ice–air two-way interactions through surface flux changes that exist in the FC run but absent in the FI run. In addition, any delayed effect from the cold season on the warm-season SST and other fields should have been already included in these differences.

### ENSO definition

We used the Niño3.4 index to quantify ENSO activity (amplitude and frequency) and the Niño3 and Niño4 indexes to measure ENSO diversity. The Niño3.4 index was defined as the monthly SST anomaly averaged within 5°S to 5°N and 170° to 120°W. The Niño3 and Niño4 indexes were similarly defined but averaged over the regions of 5°S to 5°N, 150° to 90°W and 5°S to 5°N, 160°E to 150°W, respectively. The anomaly was obtained by removing the monthly climatology and then applying a 7-year Lanczos high-pass filter with 21 weights for each month to focus on the interannual variability. Accordingly, El Niño and La Niña events were identified when the November-December-January mean Niño3.4 index was above +1 (below −1) SD in each simulation. Thus, there are 145 (80 El Niños and 65 La Niñas) ENSO events for the FC run and 147 (79 El Niños and 68 La Niñas) ENSO events for the FI run. The composites of El Niño or La Niña events were derived using similarly filtered fields. We also calculated the occurrence of CP and EP El Niño events. If the normalized Niño4 index was higher than the normalized Niño3 index, then the corresponding El Niño event was classified as CP El Niño; otherwise, it was identified as EP El Niño (*51*). We found that the ratio of the number of CP to EP events is 0.9 (38 CP events versus 42 EP events) for the FC run and 0.6 (31 CP events versus 48 EP events) for the FI run. Thus, the sea ice–air interactions increase the ratio of CP to EP El Niño events by about 50% in FC (relative to FI), although there is little change in total El Niño occurrence (80 events versus 79 events).

### ENSO growth rate and feedback

To investigate the mechanisms of the weakened ENSO amplitude caused by sea ice–air interactions, we analyzed the ENSO growth rate measured by the BJ index (*28*) in each simulation, which has been widely used to diagnose ENSO activity under different background states (*17*, *52*, *53*). The BJ index (*28*) is defined as

$$\begin{array}{c} 2\text{BJ}=-\left(a_{1}\frac{\langle ∆\bar{u}\rangle }{L_{x}}+a_{2}\frac{\langle ∆\bar{v}\rangle }{L_{y}}\right)-\alpha_{s}+\\ \mu_{a}\beta_{u}\langle -,\frac{\partial \bar{T}}{\partial x}\rangle +\mu_{a}\beta_{w}\langle -,\frac{\partial \bar{T}}{\partial z}\rangle +\mu_{a}\beta_{h}\langle \frac{\bar{w}}{H_{1}}\rangle a_{h} \end{array}$$ (1)

where $\bar{T}$ is the mean ocean mixed-layer temperature; $\bar{u}$ , $\bar{v}$ , and $\bar{w}$ are mean ocean mixed-layer zonal, meridional, and vertical velocity, respectively; 〈∙〉 denotes volume average quantities over the CEP (5°S to 5°N and 180° to 80°W), where the largest SST differences are found for both El Niño and La Niña events (see Fig. 1, A and B); *L_x_* and *L_y_* are the longitudinal and latitudinal length of the CEP box, respectively; *H*_1_ is the ocean mixed layer depth and fixed at the depth of 50 m (*H*_1_ = 50 m) in this study; *a*_1_ and *a*_2_ are estimated using SST anomalies averaged zonally and meridionally at boundaries of the CEP box and area-averaged SST anomalies over the box, respectively; α*_s_* is the thermal damping coefficient; μ*_a_* is a response of zonal wind stress to ENSO-related anomalous SSTs; β*_u_*, β*_w_*, and β*_h_* are responses of ocean surface zonal current, ocean upwelling, and zonal TH slope to anomalous zonal wind stress, respectively; *a_h_* is a response of ocean subsurface temperature to thermocline depth anomalies. More details about Eq. 1 can be found in (*28*).

On the basis of Eq. 1, the linear ENSO growth rate can be estimated as the sum of, from left to right, the mean current damping (CD) effect, the thermodynamic damping (TD) effect, the ZA feedback, the EK feedback, and the TH feedback. A higher (lower) ENSO growth rate (or BJ index) corresponds to stronger (weaker) ENSO activity with larger (smaller) amplitude. As shown in Fig. 1G, CD and TD are negative terms that are unfavorable for ENSO growth. The three positive dynamical feedback terms (i.e., the ZA, EK, and TH feedbacks) facilitate ENSO growth, and they depend on the climatological background states, the atmospheric response to ENSO-related SST anomalies, and the ocean response to anomalous wind stress (*28*). Specifically, the ZA feedback is associated with anomalous temperature advection by ocean anomaly currents, the EK feedback is associated with the mean vertical thermal stratification, and the TH feedback is related to ocean mixed-layer temperature sensitivity to thermocline variations (mainly controlled by the mean thermocline depth).

### Surface energy balance

To quantify mean change of surface net energy flux (*F*_s_) and relative contributions from surface radiation and heat fluxes induced by the sea ice–air coupling, we conducted the surface energy balance analysis (*54*). The surface energy budget equation is given by

$$F_{s}={\text{SW}}_{\text{net}}+LW_{\text{net}}+\text{SH}+\text{LH}$$ (2)

where SW_net_ is surface net SW radiation, LW_net_ is surface net LW radiation, SH is sensible heat flux, and LH is latent heat flux. Here, both SW_net_ and LW_net_ are defined as upward minus downward radiation, and all the radiation and heat fluxes in Eq. 2 are positive upward (i.e., the atmosphere receives heat from the ocean).

### Linear baroclinic model

We used an LBM (*55*) to verify whether the anomalous heating due to sea ice–air interactions over the northern North Pacific (mainly over the BOS) could generate the teleconnection anomaly pattern. LBM is based on atmospheric primitive equations linearized around a mean state and can simulate the steady linear response to a prescribed forcing, and it has been widely used in simulating atmospheric responses to specified diabatic heating (*55*, *56*). In this study, LBM has a resolution of T42L20 with a ~2.8° grid in the horizontal and 20 σ levels in the vertical, and the climatological JJAS mean state is derived from National Centers for Environmental Prediction (NCEP)/NCAR reanalysis (*57*).

We conducted two LBM simulations: One is forced by tropospheric warming over the high-latitude North Pacific (~40° to 60°N, 130°E to 150°W) with a maximum heating rate of about 1.6 K day^−1^ at location 1 (L1) near 52°N and 149°E (Fig. 5A), and the other one is the same but with additional tropospheric cooling over the subtropical North Pacific (~25° to 40°N, 130°E to 150°W) with a maximum heating rate of about −0.8 K day^−1^ at location 2 (L2) near 32°N and 160°E (Fig. 5D). The horizontal pattern of the heat forcing over the target region in both LBM simulations is identical to that of the JJAS-mean FC minus FI difference of air temperature (∆*T*) averaged over 1000 to 850 hPa (Fig. 4A), with heating rate being set to the ∆*T* multiplied by a factor of ten (e.g., a heating rate of 1 K day^−1^ is used for a 0.1°C ∆*T*); thus, such a heating rate pattern used in LBM only represents the spatial variations of ∆*T*, but they are not physically identical since we only focus on whether the anomalous low-level warming over the northern North Pacific could generate anomalous Rossby wave propagating into the tropics. Meanwhile, the vertical heating profile is given as a gamma function with peaks at the bottom of the model (σ = 0.995; Fig. 5, B and E) since the strongest ∆*T* induced by the sea ice–air coupling mainly occurs near the surface (and the lowest level). Both LBM simulations are integrated for 150 days, which reach a steady response after about 10 days. The LBM results are shown in Fig. 5.

### RWS and activity flux

To investigate the formation of anomalous Rossby wave excited by the sea ice–air coupling, we calculated the RWS (*58*) on a pressure level (e.g., 200 hPa as shown in fig. S9B) based on the nonlinear vorticity equation, which is given by

$$\text{RWS}=-\nabla ∙\left(f+\zeta \right)V_{\chi }=-\left(f+\zeta \right)D-V_{\chi }∙\nabla \left(f+\zeta \right)$$ (3)

where *f*, ζ, and their sum (*f* + ζ) are the planetary vorticity (or the Coriolis parameter), relative vorticity, and absolute vorticity, respectively, **V**_χ_ is the divergent wind, and *D* is the horizontal divergence. On the basis of Eq. 3, the RWS is composed of the vortex stretching term due to local divergence/convergence [i.e., −(*f* + ζ)*D*] and the absolute vorticity advection term induced by large-scale divergent flow [i.e., −**V**_χ_ ∙ ∇ (*f* + ζ)] (fig. S9, C and D). Given that *f* is always positive in the Northern Hemisphere and is normally an order of magnitude larger than ζ for large-scale circulation, the sign of the vortex stretching term is mainly determined by *D*; that is, a horizontal divergence (convergence) would cause the vortex squeezing (stretching) to increase negative (positive) vorticity, thus leading to a negative (positive) RWS.

We also calculated the horizontal components of the stationary Rossby wave activity flux (**W**) (*59*), which is defined as

W=p cosϕ2∣U∣Ua2cos2ϕ[(∂ψ′∂λ)2−ψ′∂2ψ′∂λ2]+Va2cosϕ[∂ψ′∂λ∂ψ′∂ϕ−ψ′∂2ψ′∂λ∂ϕ],Ua2cosϕ[∂ψ′∂λ∂ψ′∂ϕ−ψ′∂2ψ′∂λ∂ϕ]+Va2[(∂ψ′∂ϕ)2−ψ′∂2ψ′∂ϕ2] (4)

where *a* is Earth’s radius and ϕ and λ are latitude and longitude, respectively. For the CESM1 runs, *U* and *V* are the climatological JJAS-mean zonal and meridional components of horizontal wind averaged over years 1 to 500 based on the FI run, respectively, and ψ′ is the JJAS-mean difference of 200 hPa (thus, *p* = 200 hPa/1000 hPa) stream function between the FC and FI runs during years 1 to 500. For the LBM runs, *U* and *V* are the similar climatological horizontal winds obtained from NCEP/NCAR reanalysis, and ψ′ is the JJAS-mean stream function response in each runs. For the multidecadal differences in 20CRv3 reanalysis, *U* and *V* are the climatological horizontal winds averaged over P2, and ψ′ is the P1 minus P2 difference of detrended JJAS-mean stream function anomalies.

### Statistical analysis

We used Student’s *t* tests to test whether the mean state or composite differences are statistically significant based on a 5% significance level. The significance of the difference of each term in the ENSO grow rate equation (see Eq. 1) was tested on the basis of a resampling technique: we reconstructed new *X* and *Y* of length *N* (= 500 years) by randomly sampling the data from the original time series *X* from the FC run and *Y* from the FI run and then calculated the ENSO growth rates (including each term) and their difference using the randomly reconstructed new sample *X* and *Y* of the variables required in Eq. 1. We repeated this resampling procedure for 10,000 times to create a difference distribution that could occur by chance, and the 2.5th and 97.5th percentile values of this distribution were defined as the lower and higher confidence bounds, respectively, for a 5% significance level based on a two-tailed test.
