# Supplementary material for: Arctic sea ice–air interactions weaken El Niño–Southern Oscillation
Source: Sci Adv. 2024 Mar 29;10(13):eadk3990. doi: 10.1126/sciadv.adk3990 (PMC10980277; doi:10.1126/sciadv.adk3990)
Supplement: Supplementary file 1 — Figs. S1 to S9 [file sciadv.adk3990_sm.pdf]

Science Advances
AAAS

Supplementary Materials for

Arctic sea ice–air interactions weaken El Niño–Southern Oscillation

Jiechun Deng and Aiguo Dai

Corresponding author: Jiechun Deng, jcdeng@nuist.edu.cn; Aiguo Dai, adai@albany.edu

Sci. Adv. **10**, eadk3990 (2024)
DOI: 10.1126/sciadv.adk3990

This PDF file includes:

Figs. S1 to S9

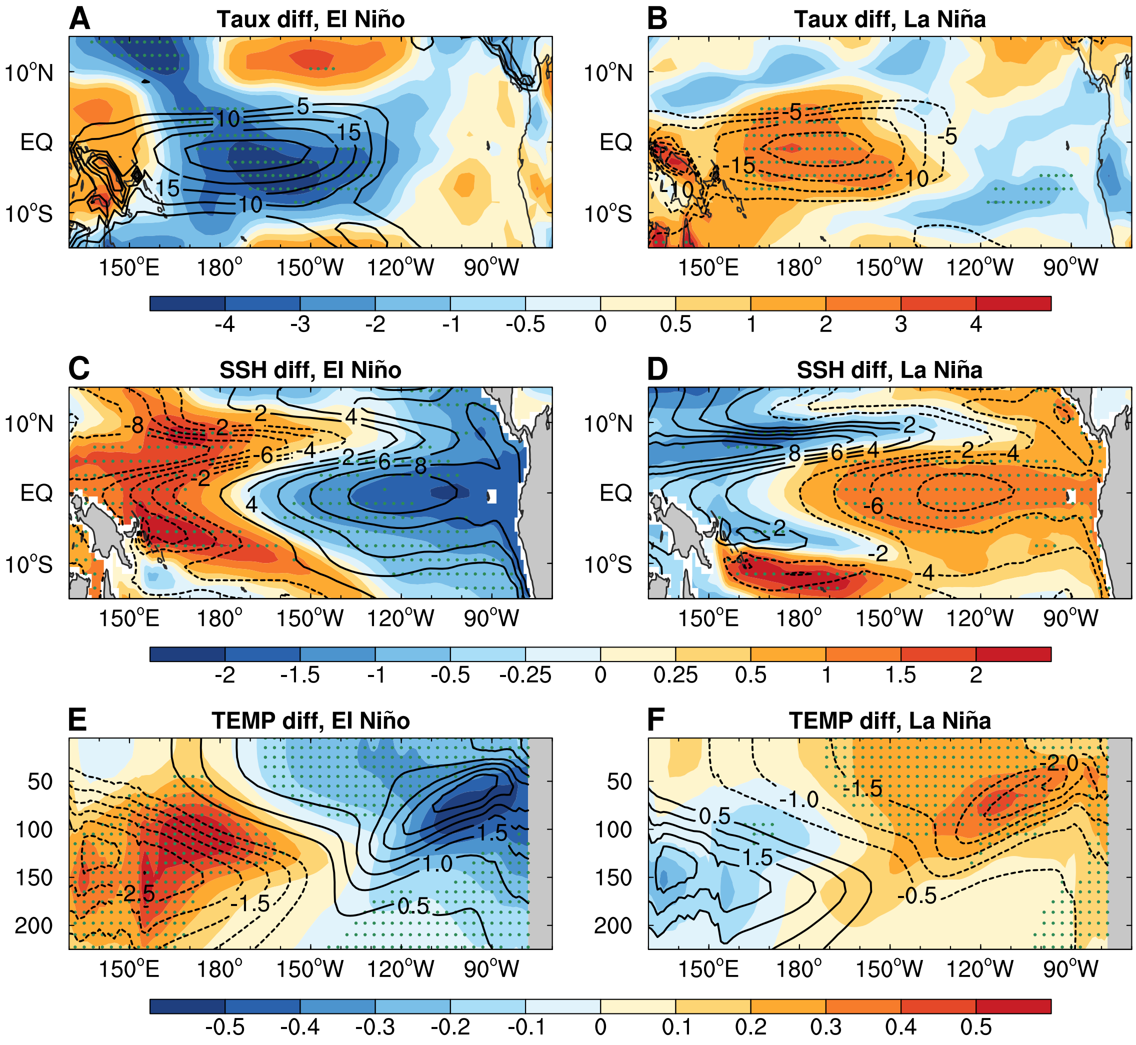

Fig. S1. El Niño-Southern Oscillation (ENSO) related difference induced by Arctic sea ice-air interactions in CESM1 model. The difference of the 7-year high-pass filtered October-February (ONDJF)-mean anomalies in (**A** and **B**) zonal wind stress (τ_x; shading, 10^{-3} N m^{-2}) and (**C** and **D**) sea surface height (SSH, used as a proxy of thermocline here; shading, cm) over the tropical Pacific averaged over (A and C) El Niño and (B and D) La Niña events between the CESM1 fully coupled (FC) and fixed ice (FI) runs during years 1–500. (**E** and **F**) Same as (A and B), but for the similarly filtered ONDJF-mean subsurface temperature (TEMP; shading, °C) anomalies above the depth of 225m averaged over the equatorial Pacific (5°S–5°N) as a function of longitude and depth. The contours show the composite anomalies from the FC run (solid and dashed are for positive and negative values, respectively, and the zero contour omitted for clarity), and the stippling indicates the difference is statistically significant at the 5% level based on a Student's *t* test.

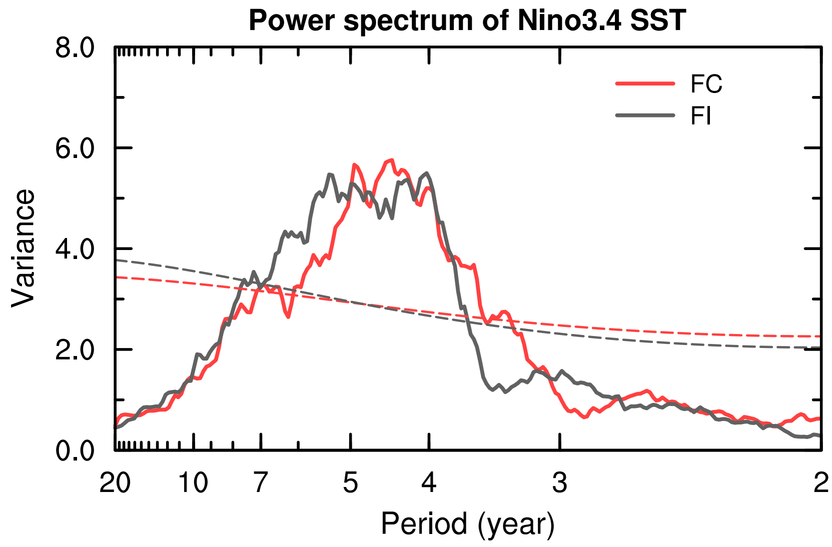

Fig. S2. Power spectrum of standardized Niño3.4 index. Power spectrum of the standardized ONDJF-mean sea surface temperature (SST) anomalies averaged over the Niño3.4 region from the CESM1 FC (red) and FI (gray) runs. The dashed lines are for the 95% confidence bound.

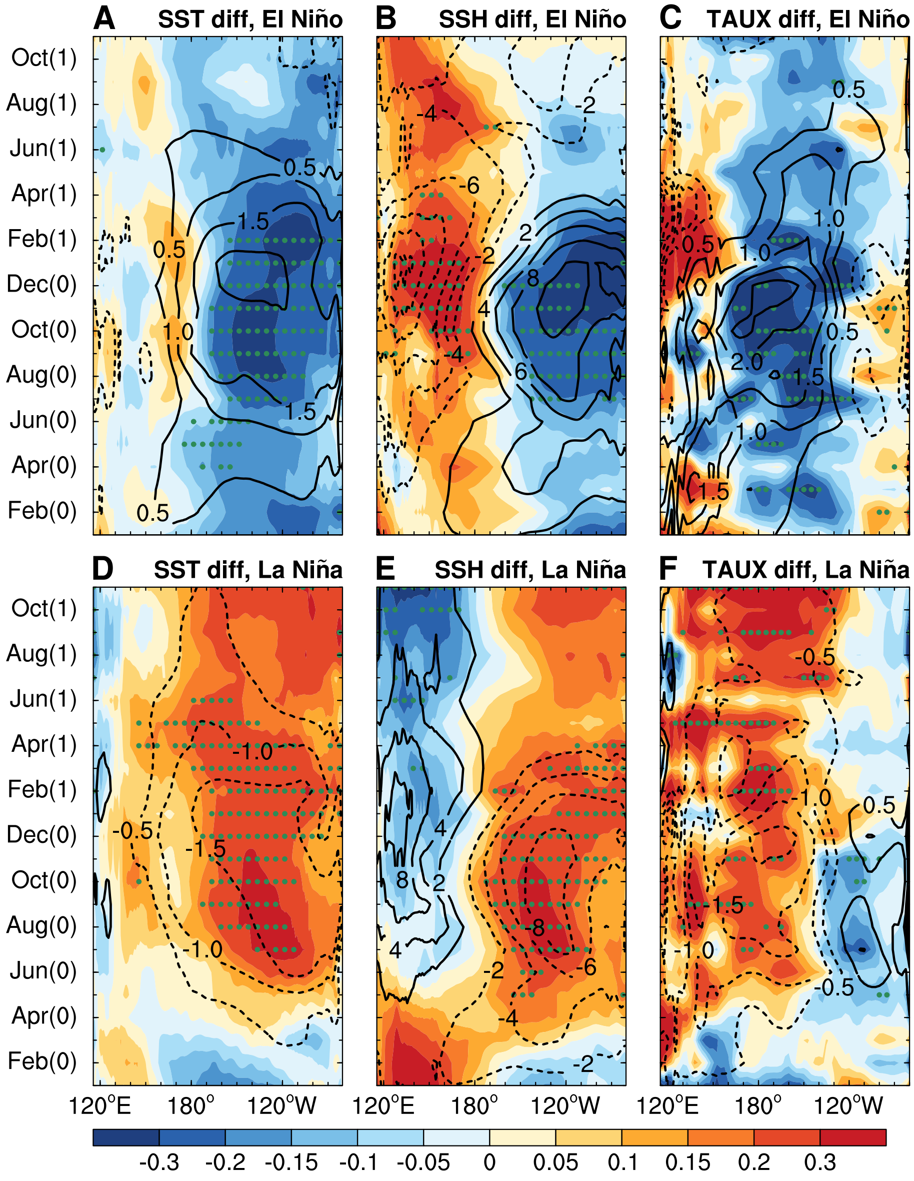

Fig. S3. CESM1-simulated seasonal evolutions of ENSO-related difference induced by Arctic sea ice-air interactions. The FC-minus-FI difference of the 7-year high-pass filtered equatorial Pacific (5°S–5°N) anomalies in (**A** and **D**) SST (shading, °C), (**B** and **E**) SSH (shading, in cm multiplied by 0.2 to use the same color table), and (**C** and **F**) τ_x (shading, 10^{-2} N m^{-2}) from the previous January [Jan(0)] to the next November [Nov(1)] averaged over (A to C) El Niño and (D to F) La Niña events during years 1–500. The contours show the composite anomalies from the FC run (solid and dashed are for positive and negative values, respectively, and the zero contour omitted for clarity). The stippling indicates the difference is statistically significant at the 5% level based on a Student's *t* test.

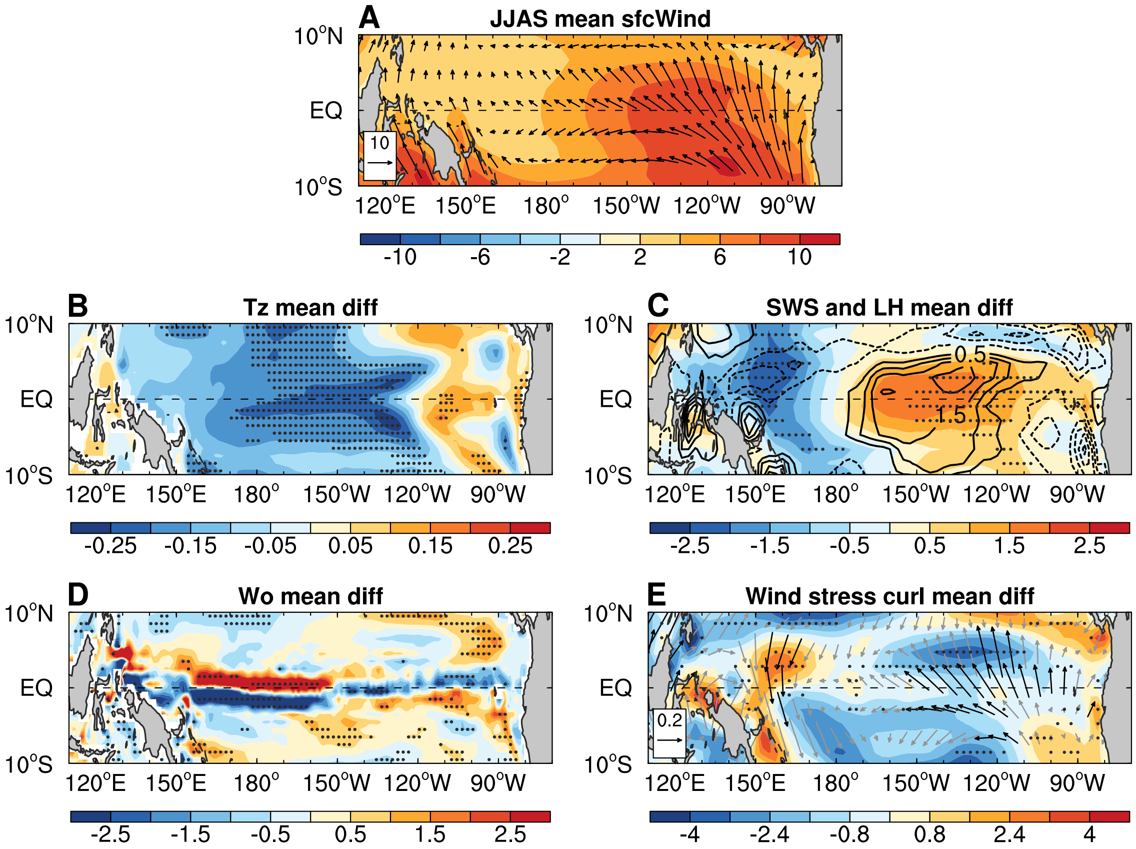

Fig. S4. CESM1-simulated climatological mean state and difference over the tropical Pacific from June to September (JJAS). (**A**) Spatial distribution of JJAS-mean surface winds (vectors, m s^{-1}) and wind speed (SWS; shading, m s^{-1}) in the CESM1 FC run during years 1–500. The FC-minus-FI differences of JJAS-mean (**B**) upper ocean stratification (measured by the difference between the mean ocean temperature over the upper 50 m and the temperature at the depth of 100 m, °C), (**C**) SWS (shading, 10^{-1} m s^{-1}) and latent heat flux (LH; contour with solid for positive and dashed for negatives; W/m^2, positive upward), (**D**) upper-50m ocean vertical velocity (Wo, 10^{-7} cm s^{-1}), and (**E**) surface wind stress (vectors, 10^{-2} N m^{-2}) and wind stress curl (shading with nine-point smoothed and multiplied by a factor of -1 in the Southern Hemisphere, 10^{-9} N m^{-3}) during years 1–500. The black vectors in (E) and the stippling in all panels indicate the difference is statistically significant at the 5% level based on a Student's *t* test.

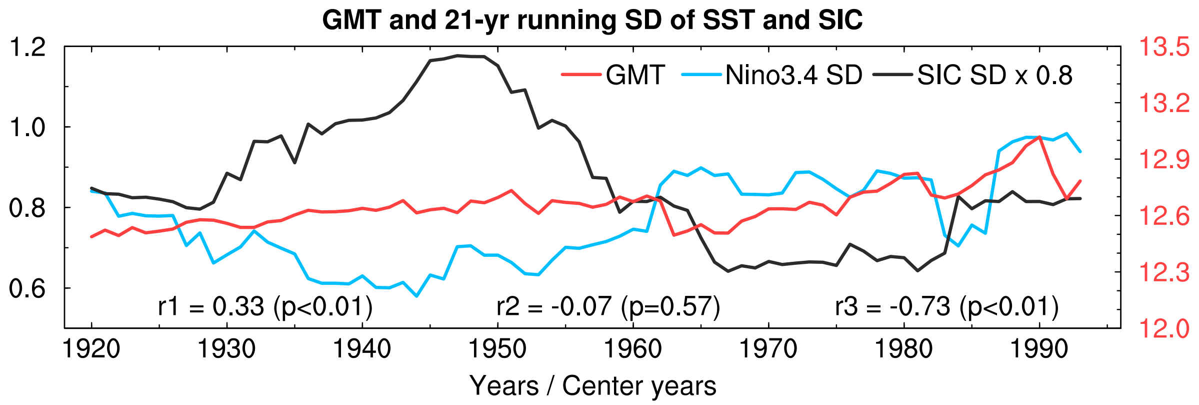

Fig. S5. Multidecadal relationship between ENSO activity and sea-ice cover (SIC) variability based on observations and reanalysis. The 21-yr running standard deviation (SD) of the 7-year high-pass filtered anomalies (with the forced component removed; see Materials and Methods) of ONDJF-mean Niño3.4 index (blue, °C; left *y* axis) and JJAS-mean SIC (black, % of area; multiplied by a factor of 0.8 to use the same left *y* axis) averaged over the Bering and Okhotsk Seas (BOS) region and the time series of ONDJF-mean global-mean surface air temperature (GMT; red, °C; right *y* axis) averaged over 25 historical runs from 25 CMIP6 models during 1920–1993. The correlation coefficients (*r*) with *P* values at the bottom are, from left to right, between GMT and SD of Niño3.4 index (*r*1), between GMT and SD of SIC (*r*2), and between SD of SIC and SD of Niño3.4 index (*r*3). Note the *x*-axis is center years for the SD time series; for example, the starting (ending) point is for the SD over 1910–1930 (1983–2003) centered at year 1920 (1993).

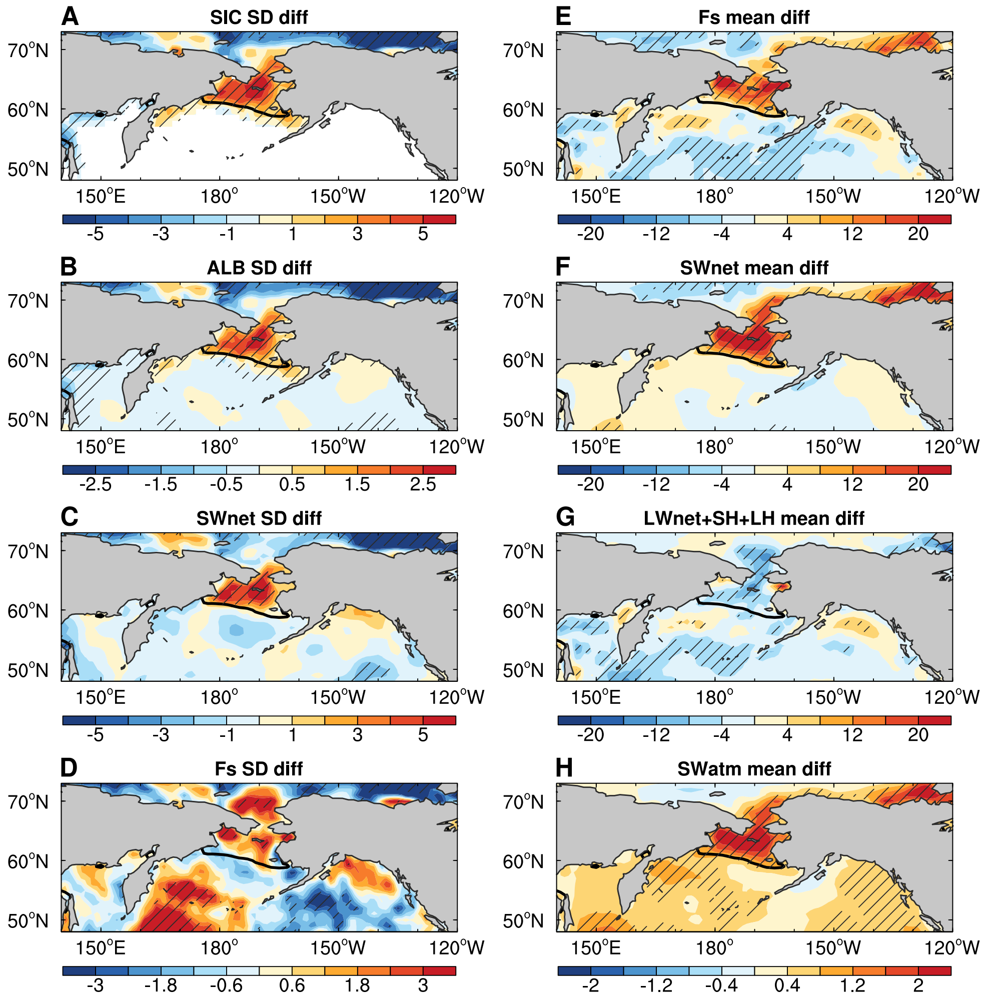

Fig. S6. Multidecadal changes of climate variability and mean state over the Pacific sector of the Arctic during JJAS based on observations and reanalysis. (**A** to **D**) The multidecadal differences of the SD of the 7-year high-pass filtered JJAS-mean anomalies (with the forced component removed; see Materials and Methods) of (A) SIC (shading, % of area), (B) surface albedo (shading, 10^{-2}, unitless), (C) surface net shortwave radiation (SW_{net}; shading, W m^{-2}), and (D) surface net energy (*Fs*; shading, W m^{-2}) over the northern North Pacific between 1921–1960 (P1) and 1971–2000 (P2) (i.e., P1 minus P2). (**E** to **H**) The P1-minus-P2 differences of the JJAS-mean anomalies (with the forced component removed) of (E) *Fs* (shading, W m^{-2}, positive upward), (F) SW_{net} (shading, W m^{-2}, positive upward), (G) surface net longwave radiation plus sensible and latent heat fluxes (LW_{net}+SH+LH; shading, W m^{-2}, positive upward), and (H) shortwave radiation absorbed by the atmosphere (SW_{atm}; shading, W m^{-2}). All surface and atmospheric fields are from 20CRv3. The black contours in all panels denote the climatological JJAS-mean SIC edge (for SIC=5%) over P1. The hatching indicates the difference is statistically significant at the 5% level based on an *F*-test in (A) to (D) and a Student's *t* test in (E) to (H).

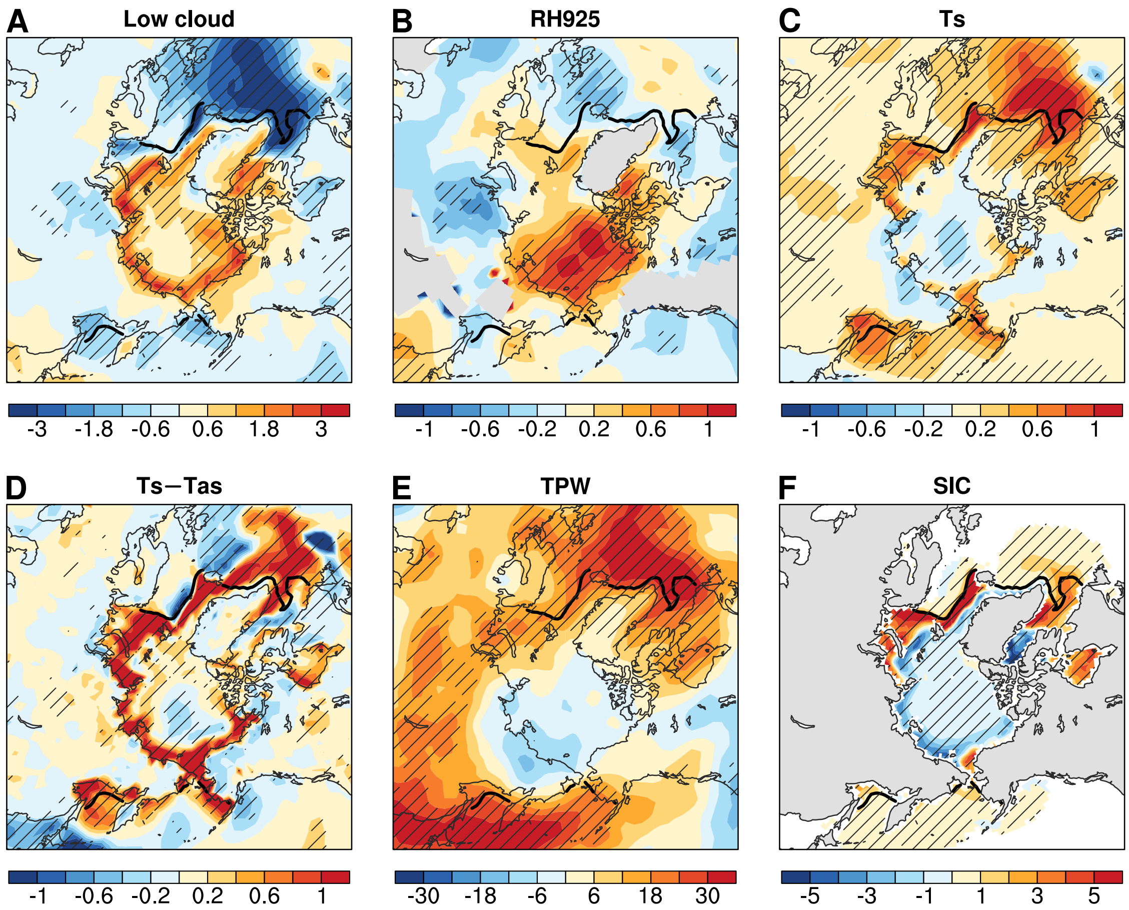

Fig. S7. CESM1-simulated Arctic mean state difference during JJAS. The FC-minus-FI differences of JJAS-mean (**A**) low cloud amount (%), (**B**) relative humidity (RH, %) at 925hPa, (**C**) surface temperature (Ts, °C), (**D**) surface-air temperature contrast (i.e., Ts minus Tas; 10^{-1} °C), (**E**) total precipitable water (TPW, 10^{-2} kg m^{-2}), and (**F**) SIC (% of area) north of 50°N during years 1–500. The black contour denotes the climatological JJAS-mean SIC edge (for SIC=5%) in the FC run. The hatching indicates the difference is statistically significant at the 5% level based on a Student's *t* test.

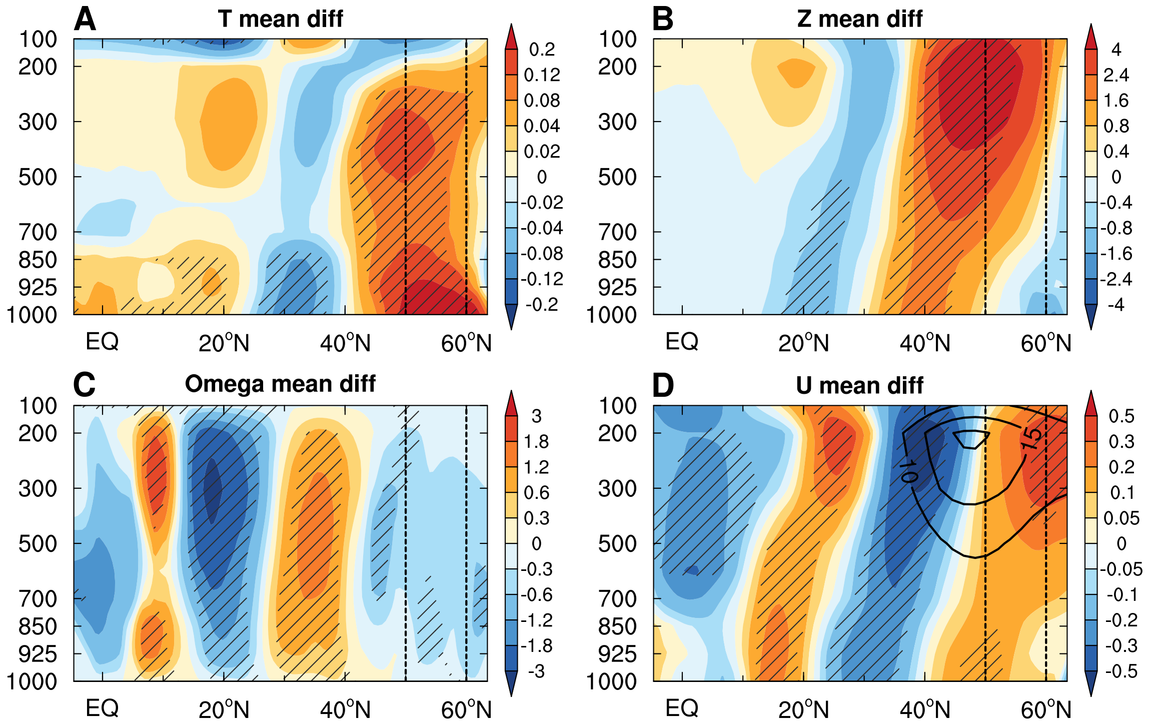

Fig. S8. Vertical structure of temperature and circulation differences during JJAS. The latitude-height section of the JJAS-mean differences of (**A**) air temperature (T; °C), (**B**) geopotential height (Z; gpm), (**C**) vertical velocity (ω; 10^{-3} Pa s^{-1}, positive for downward motion), and (**D**) zonal wind (U; m s^{-1}) averaged over 140°E–180° between the CESM1 FC and FI runs (i.e., FC minus FI) during years 1–500. The black contours in (D) denote the climatological JJAS-mean U from the FC run. The dashed lines represent the northern North Pacific over 50°–60°N. The hatching indicates the difference is statistically significant at the 5% level based on a Student's *t* test.

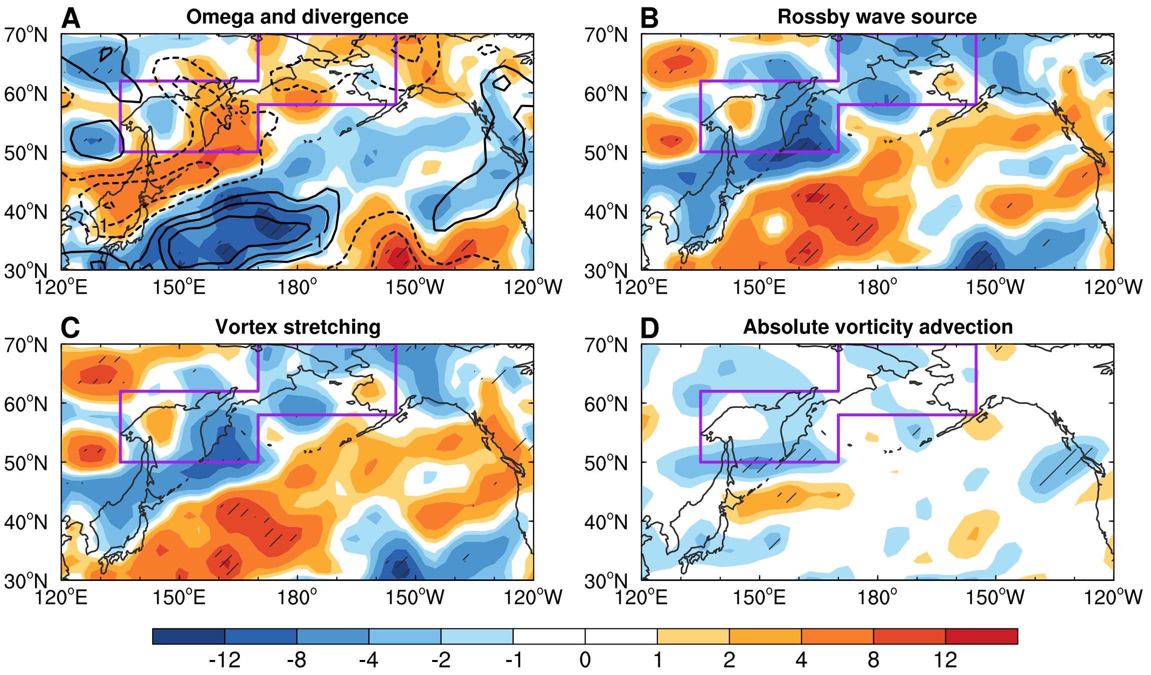

Fig. S9. Differences of Rossby wave source (RWS) and its components induced by sea ice-air interactions during JJAS in CESM1. The FC-minus-FI difference of JJAS-mean (**A**) 200hPa divergence (shading, 10^{-8} s^{-1}) and 500hPa ω (10^{-3} Pa s^{-1}, contours with an interval of 0.5 and the zero contour omitted for clarity) and (**B**) 200hPa RWS and its (**C**) vortex stretching term and (**D**) absolute vorticity advection term (10^{-12} s^{-2}; see Materials and Methods) over the North Pacific north of 30°N during years 1–500. A nine-point smoothing was applied to all panels. The hatching indicates the difference is statistically significant at the 5% level based on a Student's *t* test. The outlined area is the BOS region as in Fig. 3.
